# Aromatase and 5-alpha reductase gene expression: modulation by pain and morphine treatment in male rats

**DOI:** 10.1186/1744-8069-6-69

**Published:** 2010-10-26

**Authors:** Anna Maria Aloisi, Ilaria Ceccarelli, Paolo Fiorenzani, Melinda Maddalena, Alessandra Rossi, Valentina Tomei, Giuseppina Sorda, Barbara Danielli, Michele Rovini, Andrea Cappelli, Maurizio Anzini, Antonio Giordano

**Affiliations:** 1 Department of Physiology, Neuroscience and Applied Physiology Unit, University of Siena, Via Aldo Moro 2, 53100 Siena Italy; 2 Department of Human Pathology and Oncology, University of Siena, Strada delle Scotte 6, 53100 Siena Italy; 3 San Carlo Clinic, Via Ospedale, 21, 20037 Paderno Dugnano (Milano), Italy; 4 Dipartimento Farmaco Chimico Tecnologico, Università degli Studi di Siena, Via A. Moro, 53100 Siena, Italy; 5 Pain Neurophysiology and Therapy Centre, IRCCS Fondazione Salvatore Maugeri, Via S. Maugeri, 10 27100 Pavia, Italy; 6 Sbarro Institute for Cancer Research and Molecular Medicine and Center of Biotechnology, Temple University Philadelphia PA USA

## Abstract

**Background:**

The steroid hormone testosterone has been found to be greatly reduced by opioids in different experimental and clinical conditions. The purpose of this study on male rats was to determine the effects of a single injection of morphine (5 mg/Kg) on persistent pain (formalin test) and the single or combined effects on p450-aromatase and 5-alpha reductase type 1 mRNA expression in the brain, liver and testis. Testosterone was determined in the plasma and in the brain, morphine was assayed in the plasma.

**Results:**

In the morphine-treated rats, there were increases of 5-alpha reductase mRNA expression in the liver and aromatase mRNA expression in the brain and gonads. Morphine was detected in the blood of all morphine-treated rats even though there were no clear analgesic affects in the formalin-treated animals three hours after treatment. Testosterone was greatly reduced in the plasma and brain in morphine-treated subjects.

**Conclusions:**

It appears that morphine administration can induce long-lasting genomic effects in different body areas which contribute to the strong central and peripheral testosterone levels. These changes were not always accompanied by behavioral modifications.

## Background

It has repeatedly been shown *in vitro* and in vivo that opioids change gonadal hormones after treatment, resulting in a dramatic alteration of the hormones ratio [1,2]. In particular we have shown that testosterone (T) levels are significantly decreased by morphine independently of the duration of treatment and with clear clinical signs (fatigue, anemia) [3,4]. This clear long-lasting depressant effect has been attributed to direct inhibition of morphine at hypothalamic levels: i.e. a decrease of gonadotropin releasing hormone (GnRH) followed by a decrease of T production by the testis [5]. More recently this hypothesis was supported by evidence showing an involvement of other neural and metabolic structures in which T-catabolic enzymes were modulated by opioids, suggesting increased catabolism of this hormone able to explain its fast disappearance from the blood [6-8]. Indeed, although not related to pain, steroid hormones and opioid interactions in the brain have been extensively described [9].

T is produced by the testis but can also be obtained from other metabolic pathways present in other body structures, including the brain [10]. Its action is widespread and includes peripheral and central effects obtained with classic genomic or rapid membrane-mediated actions [11]. Testosterone can bind directly to androgen receptors (ARs) or can be aromatized to estradiol (E_2_), acting on estrogen receptors (ERs), or reduced to dihydrotestosterone (DHT), acting on ARs. These metabolites (E_2 _and DHT) play crucial roles in the organism, mediating many functions such as cognition, reproduction, feeding, muscle, blood and brain metabolism [12,13]. Thus modulation of the enzymes 5-alpha reductase and aromatase has to be considered an important gateway to these functions. The former plays an important role in testosterone conversion to DHT in androgen-dependent tissue, the latter in T aromatization to E_2_. Two 5-alpha reductase isoenzymes have been characterized in rats: type 1 and type 2, reported to play a catabolic and an anabolic role on T, respectively. In this experiment we study the mRNA expression of 5-alpha reductase type 1 (5aR-1) and p450-aromatase (AROM). AROM is primarily expressed in the hypothalamus and limbic regions of the brain. The peripheral expression of aromatase is widely distributed. Early studies on aromatization in the hypothalamus of male and female rats were reported by Naftolin [14].

Pain therapy with opioid drugs is based on their ability to bind opioid receptors in the CNS and in the periphery; in particular, through its binding to opioid receptors, morphine is known to act in several body regions from the gut to the brain [15,16]. Previous studies have clearly shown that morphine induces a long-lasting decrease of T even if treatment lasts for months or years [4]. The effect can be induced by low and subanalgesic concentrations of morphine and occurs after a few hours. We have also shown that once treatment is interrupted the levels recover in a few hours/days [3,17].

To evaluate their relative expression and modulation by morphine and pain, 5aR-1 and AROM were determined in the diencephalon, liver and testis. While the diencephalon is the key structure to be studied in view of the long-term effects that can be produced by T deficiency [18], the liver and testis are peripheral structures representing its main site of production (testis) and degradation (liver). Behaviors were recorded and analyzed during the formalin test while T was determined at the end of the test in blood and brain samples.

## Results

**Figure 1:**
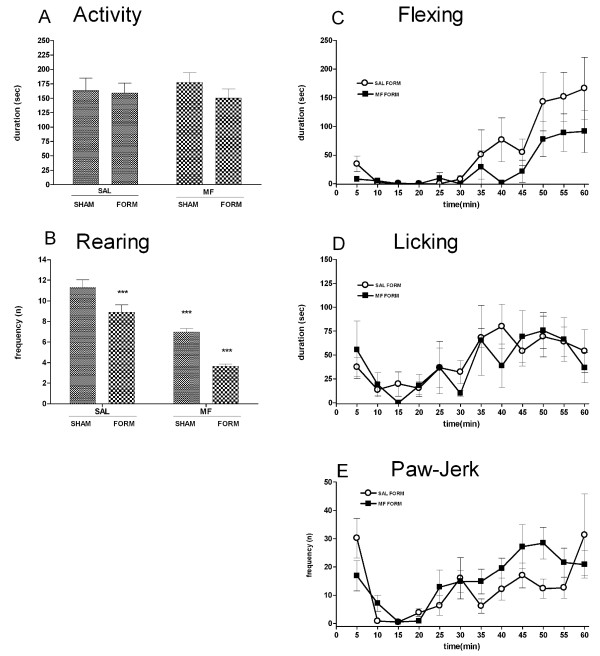
**Behaviors recorded during the formalin test (60 min)**. Spontaneous behaviors: (A) activity duration (sec) and (B) rearing frequency (n). Pain-related behaviors: (C) flexing duration, (D) licking duration and (E) paw-jerk frequency recorded in male rats treated with morphine (MOR) or saline (SAL) three hours before the formalin test in the dorsal hind paw. Data are shown as mean ± SEM.

**Figure 2:**
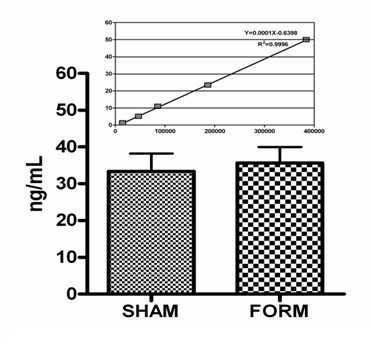
**Morphine levels in SHAM and FORM groups four hours after a single injection of morphine**. Data are shown as mean ± SEM. Insert: calibration curve for morphine. The levels were comparable between the two groups.

The behavioral analysis carried out during the formalin test showed that spontaneous behaviors were affected by morphine although recorded three hours after administration (Figure 1A, B), whereas as expected the administration of a subanalgesic concentration of morphine did not produce any significant modification in formalin-induced pain responses (Figure 1C, D, E). Morphine was present in the blood at detectable levels at the end of the experiment, four hours after treatment (Figure 2).

**Figure 3:**
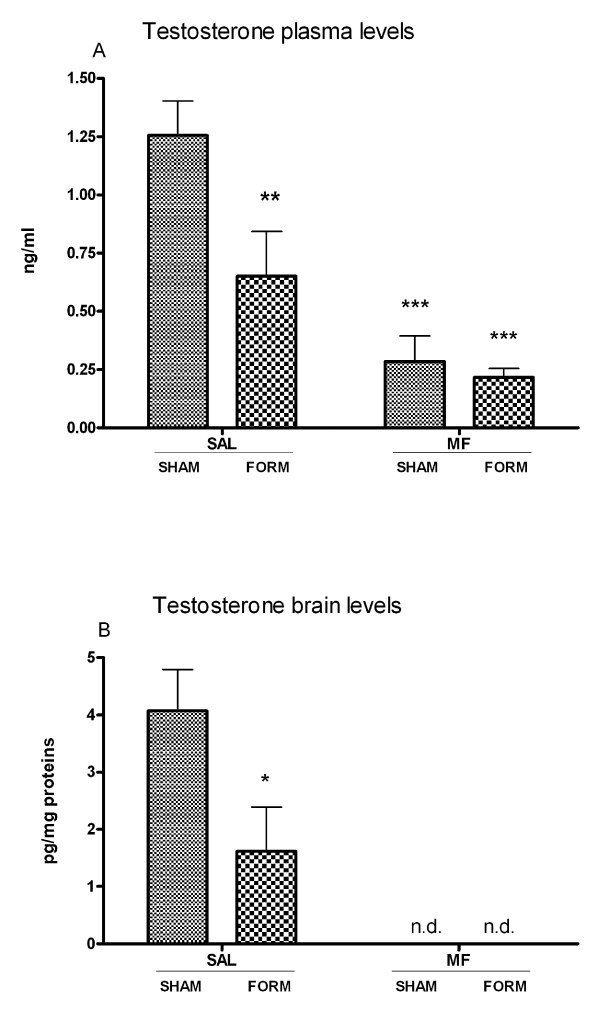
**Testosterone plasma (A) and brain (B) levels determined four hours after treatment in morphine (MOR)-treated animals and controls (SHAM); in formalin (FORM)-treated animals and controls (SHAM)**. Data are shown as mean ± SEM.

The blood testosterone levels were strongly decreased in the formalin and/or morphine-treated groups (p = 0.01 and p < 0.001, respectively) but not in the saline-treated ones (Figure 3A); testosterone was not detected in the brain in both morphine-treated groups (Figure 3B). The comparison between the two saline-treated groups (T-Student) revealed significantly lower testosterone levels (p < 0.05) in the SAL/FORM group than in the SAL/SHAM one.

### 5-alpha reductase (5aR-1) and aromatase (AROM) mRNA expression

**Figure 4:**
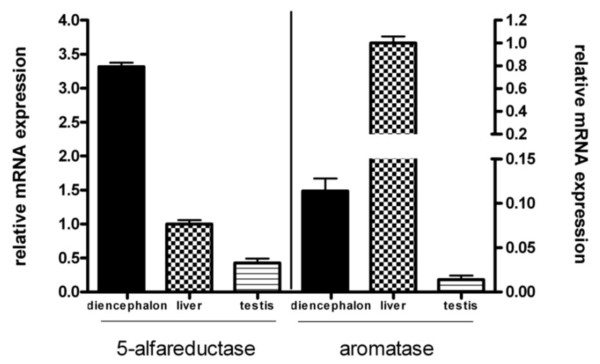
**Relative mRNA expression of 5-alpha reductase type 1 and p450-aromatase in the diencephalon, liver and testis of the control group (SAL/SHAM)**. Liver is the reference organ, indicated as 1. Data are shown as mean ± SEM.

Figure 4 reports the relative mRNA expression of 5aR-1 and AROM in the diencephalon, liver and testis in the control group. Our data indicate that 5aR-1 expression was much higher in the diencephalon than in the liver (used as reference, i.e. 1) while its expression in the testis was only half that in the liver. In contrast, AROM expression in the diencephalon was about 10% of that in the liver and the levels in the testis were very low (about 2%). The ratio among these three tissues is important because it indicates whether the stimulus used affects only their absolute activity or also changes the relative activities of the enzymes in the different tissues.

**Figure 5:**
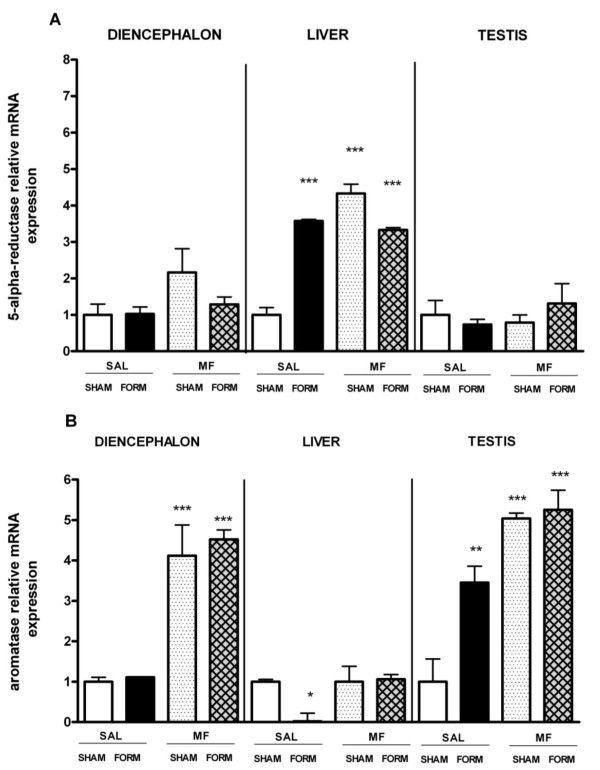
**5-alpha reductase type 1 (A) and p450-aromatase (B) mRNA expression in the diencephalon, liver and testis**. SAL/SHAM is the reference group, indicated as 1. Data are shown as mean ± SEM. Statistical significance vs SAL/SHAM *p < 0.05; **p < 0.01; ***p < 0.001.

Figure 5 shows the relative mRNA expression of 5aR-1 and AROM in the diencephalon, liver and testis in all groups. The 5aR-1 mRNA expression in the *diencephalon *differed among groups (p < 0.012); it was 100% higher in the MOR/SHAM group than in the others (p < 0.05) while in MOR/FORM the increase was smaller and not significant. In the same tissue there was an effect of Treatment (p < 0.001) on AROM expression due to its upregulation in both morphine groups (p < 0.001) compared to both saline groups; hence the expression was independent of pain.

In the *liver*, the 5aR-1 mRNA expression was increased (p < 0.001 per all) about fourfold by formalin and/or morphine treatment (p < 0.001) while the AROM expression was significantly decreased in the SAL/FORM group (p < 0.05) and unchanged in the other groups.

In the *testis*, no differences in 5aR-1 gene expression were observed among groups. In contrast the AROM mRNA expression showed a marked change in all groups when compared to the SAL/SHAM group (p = 0.0001); in SAL/FORM the increase was about twofold (p < 0.01) while in both morphine groups the increase was about fourfold (p < 0.001).

## Discussion

The main result of the present experiment is that the expression of 5-alpha reductase type 1 (5aR-1) and p450-aromatase (AROM) in the male rat brain, liver and testis was strongly affected by inflammatory pain and morphine. These changes, which differed among the tissues, were not accompanied by evident changes in pain behaviors but by a clear decrease of testosterone (T) levels in the blood and diencephalon.

The main cause of these immediate and long-lasting effects has been attributed to the inhibitory action of morphine on gonadotropin-releasing hormone secretion in the hypothalamus [see 3]. This inhibition is able to decrease gonadotropin production and then gonadal hormone secretion. However it has become clear that morphine-induced hypogonadism, observed in patients treated with this drug, is not merely centrally mediated and that other 'peripheral' active sites must be considered. Thus it was decided to study testosterone catabolic enzymes in order to indicate the changes occurring in that system. We showed upregulation of AROM activity in astrocytes *in vitro*[6] and Amini *et al*. [7] demonstrated an increase of 5-alpha reductase expression in vivo. In agreement with the previous data and with our initial hypothesis, we showed in the present experiment that a single s.c. injection of morphine changed the aromatase activity in two of the three tissues taken into account, strongly indicating an increase of the catabolic activity of testosterone at least in these tissues.

The high AROM mRNA expression observed in the diencephalon and testis suggests an increase in enzymatic activity resulting in increased metabolism of testosterone to estradiol (E_2_). The role of estrogens in the nervous system is well known; in male and female subjects, many neural circuits are modulated by these hormones. In particular, E_2 _shows close interactions (cross-talk), cross-coupling and reciprocal regulation with proteins involved in signal transduction mediated by neural growth factors [19] or neurotransmitters [20,21]. Moreover, estrogens play a role in activation of the molecular cascade involved in plastic adjustment of cellular functions inducing c-Fos expression (one of the first signs of neuronal plasticity) in the hippocampus [22], activate MAP-kinase (a growth factor) [23] and potentiate glutamate binding to N-methyl-d-aspartate (NMDA) receptors [20,21]. Thus it appears that the structural and functional changes induced by E_2 _help to increase seizure (pain or stress) susceptibility. On the other hand, estrogens were shown to *limit *neuronal damage and death, possibly by signaling through PI3K, PKC, ERK or glycogen synthase kinase 3-β [24]. In the *testis*, AROM is responsible for the transformation of androgens into estrogens using either androstenedione or T as androgenic substrates, which are aromatized to estrone (E_1_) and E_2_, respectively [25]. This activity can be explained by the need to increase E_2 _in these structures actively involved in the regulation of reproductive testis functions [26]. Interestingly, AROM expression was not affected by morphine treatment in the liver. This is important since this enzyme is the one used to metabolize codeine to morphine in the same tissue [27].

In the present study we also determined the expression of 5aR-1, the other enzyme involved in testosterone degradation, in the same tissues. The expression was affected by morphine in the diencephalon and liver but not in the testis. 5aR-1 acts on T to produce DHT. In the CNS, DHT was found to have particular organizational effects on selected neuronal populations [28]. At the cellular level, the direct role of androgens in the brain is supported by the observation that both T and DHT modify the number of branching points in preoptic neurons in culture [29], acting particularly as morphogenetic signals for the developing hypothalamic neurons containing AROM and thus influencing the plasticity and synaptic connectivity of the hypothalamic aromatase system [30]. The activational effects of DHT on cognition have been far less studied than the effects of T. It has been shown, however, that DHT is a powerful steroid in the CNS with a fourfold higher affinity to the human ARs than T [31]. Recently, Cherrier *et al*. [32] prospectively examined changes in cognition in hypogonadal men given T and older hypogonadal men given DHT; they demonstrated significant improvements in verbal memory in response to T supplementation with increased serum levels of T, DHT and E_2 _(secondary to aromatization), although the increases in spatial memory did not reach statistical significance. In older hypogonadal men, DHT treatment, with increased DHT and decreased T serum levels, resulted in a significant improvement in spatial memory. These findings are supported by research on male rats in which there was an increase in spine synapse density in the CA1 sub-field of the hippocampus after treatment with testosterone propionate (TP) or DHT [33].

An interesting point to consider are the effects induced on T and its enzymes by formalin pain per se. T levels were decreased in the brain and blood of formalin-treated animals. It should be noted that formalin pain per se decreased AROM and increased 5aR-1 expression in the liver. This appears to be an important shift in the metabolic pathways: DHT production seems to be preferred to E_2_, in line with the observation by Negri-Cesi *et al*. [34] who showed a decrease in AROM activity in hypothalamic male rat neurons exposed to DHT *in vitro*.

In our adult male rats, AROM was much more highly expressed in liver than in the diencephalon and testis; nevertheless, its physiological role is not very clear since liver is generally considered a non-steroidogenic tissue [35,36]. However the huge increase in 5aR-1 in the liver can be explained by the need to eliminate androgens and protect against excessive hormone levels; the pathway responsible for the switch from T to DHT activity involves the irreversible reaction catalyzed by this enzyme [37].

Another point is the lack of any changes in formalin-induced pain in morphine-treated animals. Therefore the morphine-induced changes in gonadal hormone metabolism would be able by themselves to completely cancel the expected analgesic effect of the morphine. This is in agreement with Nagypal *et al*. [38] who provided neuroanatomical evidence that T-induced brain activation overlaps with opiate-responsive regions; T may activate opiate-responsive neurons directly or indirectly via metabolites to facilitate reinforcement. It appears that once morphine enters the body it acts to increase the production of these metabolites. However the low T levels in both humans and experimental animals treated with morphine suggest that this effect would be useful in the short term (i.e. during an acute stress with the release of endogenous opioids), whereas the longer lasting effects obtained with pain therapy would not be useful and could initiate a series of negative effects well known to pain therapists.

## Conclusions

In the present study we determined the gene expression of the two main catabolic enzymes of T and we showed that morphine treatment significantly changes 5aR-1 and AROM mRNA expression in different body regions. There was a significant increase in the expression of one or both of these enzymes in the brain as well as in the liver and testis, suggesting a pro-metabolic action of morphine. Hence T is not only produced to a lesser degree because of the well known opioid effects on gonadotropin secretion, it is also metabolized to a greater degree, with the net result of very low blood and brain levels. At present this evidence cannot be considered negative since both E_2 _and DHT can have beneficial effects on neurons, although detrimental effects have also been described [39]. The very low (n.d.) levels of T in the brain suggest that when T levels are very low in the blood the possibility of this hormone being present in the brain is very low [8]. We have recently shown *in vitro* that supplementation of astrocytes with exogenous T strongly increases the testosterone cell content [6]. Thus, although the production of T in the CNS is possible, the exogenous supplementation of this hormone would play an important role in maintaining acceptable levels and functions.

## Methods

### Subjects

Thirty-five gonadally intact male Sprague-Dawley rats (Harlan, Italy), weighing 320-400 g, were used. The animals were housed in groups (4 per cage) in plastic-bottomed cages with sawdust bedding and kept at room temperature 21 ± 1 C°, relative humidity 60 ± 10% and a 12/12 h light/dark cycle. They received food and water *ad libitum*. Lights went off at 07:00 a.m. and testing was carried out between 09:30 a.m. and 12:30 p.m. during the dark phase, the active period of rodents. The experimental procedures were pre-approved by the Ethics Committee of the University of Siena. In all experiments, attention was paid to the regulations for handling laboratory animals of the European Communities Council Directive (86/609/EEC) and the Ethical Guidelines for investigation of experimental pain in conscious animals issued by the ad-hoc Committee of the International Association for the Study of Pain [40]. Particular efforts were made to minimize animal suffering and to reduce the number of animals used.

### Materials and Reagents

All chemicals were reagent or HPLC grade from Sigma-Aldrich (St. Louis, MO) with the exception of the morphine (S.A.L.A.R.S., Como, Italy) and morphine-d_3 _from Lipomed AG (Arlesheim, CH).

### Groups

The animals were randomly assigned to four experimental groups:

•SAL/SHAM (n = 11, rats treated with saline and sham-injected in the formalin test),

•SAL/FORM (n = 8, rats treated with saline and formalin-injected in the formalin test),

•MOR/SHAM (n = 8, rats treated with morphine and sham-injected in the formalin test),

•MOR/FORM (n = 8, rats treated with morphine and formalin-injected in the formalin test).

### Treatment

Treatment consisted of injection of morphine (5 mg/Kg) or saline (NaCl 0.9%) into the subcutaneous tissue of the back while the animals were gently restrained. A mean volume of 220 μl was injected subcutaneously (s.c.) into each animal. Immediately after the injection the rats were returned to their home cage to rejoin the original group.

### Formalin test (FT)

Three hours after the morphine or saline injection the rats were randomly assigned to SHAM or FORM groups. FORM animals received dilute formalin (10%, 50 μl) s.c. in the right dorsal hind paw. Sham animals were merely pricked with the syringe needle, without injection of any substances. The rat was then placed in the Open Field apparatus and its behavior was recorded for 60 min. To determine pain intensity and to verify the behavioral effects of treatment, pain-related and spontaneous behaviors were considered:

a) pain-related behaviors (formalin-induced responses): licking duration (time spent licking the injected foot); flexing duration (time spent with the leg held off the floor, flexed close to the body); paw-jerk frequency (number of phasic flexions of the leg).

b) spontaneous behaviors: rearing frequency (number of times the animal stood on its fore limbs); activity duration (time spent sniffing and looking around the environment, even the time spent washing or scratching the face or body).

### Parameters

At the end of the formalin test the rats were anesthetized with sodium pentobarbital, the abdomen was opened and blood was collected from the abdominal vein in EDTA-added syringes. The animals were intracardially perfused with phosphate buffered saline (PBS, about 200 ml) for exsanguinations of the CNS. Then the diencephalon, the gonads and part of the liver were dissected and frozen till gene expression determination. The blood was centrifuged and the plasma was divided into aliquots and immediately frozen till morphine and hormonal determinations.

### Morphine determination

Solid phase extraction (SPE) was performed with disposable ISOLUTE^® ^non-polar SPE sorbents (Biotage) to extract acidic, basic or neutral drugs from biological fluids using a non-polar (hydrophobic) retention mechanism together with a Vac-Elut setup. Briefly, the cartridges were conditioned with 2 ml of MeOH and 2 ml of borate buffer (pH 9; 50 mM) using vacuum aspiration. 250 μl of plasma were added to 500 μl of borate buffer (pH 9; 50 mM). This solution was loaded onto the cartridges and slowly drawn through them. The cartridges were sequentially washed with 1 ml of water/methanol (95:5) and twice with 500 μl of acidic MeOH (formic acid 2%, v/v) using vacuum aspiration. Finally, the sample was dried under N_2 _and reconstituted with 250 μl of solution used for the mobile phase LC-MS/MS analysis. Before SPE, an internal standard (IS) of morphine-d_3 _was added to all plasma samples at a concentration of 20 ng/ml.

The analysis was performed with a Varian chromatographic instrument (Varian Inc.) operated by the Varian MS Workstation software system Control Vers 6.9, consisting of a binary pump (212-LC) detector connected to a Varian triple quadrupole (LC-MS 320/MS) with ESI ionization source. The sample for the chromatographic analysis was obtained under the conditions described above. For the chromatographic analysis, a Pursuit C18 column (3 μm 100 × 2 mm) (Varian) was used. The mobile phase consisted of: (A) 1% formic acid in water plus 3 mM ammonium acetate; and (B) HPLC grade methanol. A linear gradient from 90% A to 10% A in 8 min was used and after 2 min the gradient returned to 90% A for 5 min. The column temperature was maintained at room temperature. The injection volume was 10 μl; run time was 16 min; flow rate was 0.2 ml/min. The mass spectrometer was operated in the positive mode. Quantification was performed using selected reaction monitoring (SRM) of the transitions of the precursors to the product ions as follows: mass-to-charge ratio (m/z) 286 → 201 for morphine and (m/z) 289 → 201 for morphine-d_3 _(internal standard (IS)), with a scan time of 0.3 s per transition. The tuning parameters were optimized for morphine by infusing a solution containing 1 μg/ml of analyte at a flow rate of 10 μl/min into the mobile phase (0.2 ml/min) using a post-column "T" connection. The optimal MS parameters obtained were as follows: the spray voltage was 5000 V with a source CID voltage of 10 eV, the heated capillary was 350°C. Nitrogen was used as the sheath gas (45 psi) and auxiliary gas (19 psi). Argon was used as the collision gas at a pressure of approximately 1.80 mtorr. The optimized collision energy chosen for morphine was 19 eV. The concentration of morphine in the samples was calculated in comparison with the peak area of morphine-d_3 _(IS) and each sample was analyzed in triplicate. For all samples the mean, standard deviation and coefficient of variation (always less than 15) were also calculated.

### RNA extraction and real-time quantitative reverse transcription PCR

Total RNA from liver, diencephalon and testis was extracted and purified with a NucleoSpin RNA II kit (Macherey-Nagel GmbH&Co KG, Germany) following the manufacturer's instructions. The RNA concentration was measured with a NanoDrop ND-100 spectrophotometer. qRT-PCR was performed to monitor the gene expression levels of 5-alpha reductase type 1 and p450-aromatase. Five hundred nanograms of RNA were reverse transcribed with the Transcriptor High Fidelity cDNA Synthesis kit (Roche, Mannheim, Germany) using oligo (dT) primers (Roche). One microliter of the cDNA was amplified using Opticon II (MJ Research, Waltham, MA) and the SYBR Green PCR Master Mix (Applied Biosystems, Warrington, UK), following the manufacturer's instructions. Forty PCR cycles were performed using an annealing temperature of 60°C for all the genes tested. Primers were specifically designed between two adjacent exons (AutoPrime program) and the sequences used in this study were: for 5-alpha reductase, CGTCCTGCTGGCTATGTTTC (forward), GAAGGCCAAGACAAAGGTGA (reverse); for p450-aromatase, CGAGATCGAAATTCTGGTGGAAAAG (forward), TGCAAAATCCTACAGTCTTCCAGTT (reverse); for cyclophilin (housekeeping gene), ACACGCCATAATGGCACTGG (forward), ATTTGCCATGGACAAGATGCC (reverse).

### Normalization of real-time PCR quantification

mRNA levels for 5-alpha reductase and P450-aromatase (Ct) were normalized to cyclophilin by subtracting the Ct value of the reference gene (cyclophilin) from the Ct value of the samples (ΔCt = Ct _sample_-Ct _reference_). The relative expression of the target gene to a calibrator is quantified using 2^-ΔΔCt ^[41]. The calibrator was defined as liver tissue the first time and as diencephalon or liver or testis for the other comparative analysis. Finally the relative expression was determined by subtracting the ΔCt _calibrator _(ΔCt _calibrator _= Ct _calibrator_-Ct _reference_) from the ΔCt value (ΔΔCt = ΔCt _calibrator_-ΔCt).

### Testosterone determination in blood and brain

Blood and brain testosterone levels were extracted and determined by radioimmunoassay (RIA) according to different methods [42-44], revised and adapted [17]. Samples were assayed in duplicate.

### Statistical analysis

Data were expressed as mean ± SEM. ANOVA was applied with the factors Treatment (two levels: SAL, MOR) and Pain (two levels: SHAM, FORM), followed by Fisher's protected least significant difference (PLSD) post hoc test as appropriate. T-Student was used to compare morphine concentrations in the two morphine-treated groups. The criterion for statistical significance was p < 0.05.

## Competing interests

The authors declare that they have no competing interests.

## Authors' contributions

AMA, MA and AG conceived and supervised the project and edited the manuscript.

IC, PF, MM, AR, VT, GS, BD, MR, AC participated in the experimental process and data analysis.

All authors contributed to data interpretation and have read and approved the final manuscript.
